# Analyzing Latent Burnout Profiles in a Sample of Spanish Nursing and Psychology Undergraduates

**DOI:** 10.3390/healthcare12040438

**Published:** 2024-02-08

**Authors:** Raimundo Aguayo-Estremera, María José Membrive-Jiménez, Luis Albendín-García, José L. Gómez-Urquiza, José Luis Romero-Bejar, Emilia Inmaculada De la Fuente-Solana, Gustavo R. Cañadas

**Affiliations:** 1 Department of Psychobiology and Methodology in Behavioral Sciences, Complutense University of Madrid, Campus de Somosaguas, 28223 Pozuelo de Alarcón, Spain; r.aguayo@ucm.es; 2 Faculty of Health Sciences, University of Granada, Avda. Ilustración 60, 18016 Granada, Spain; mjmembrive@ugr.es; 3 Casería de Montijo Health Center, Granada Metropolitan District, Andalusian Health Service, Calle Joaquina Eguaras nº 2, Edificio 2 1ª Planta, 18013 Granada, Spain; luis.albendin.sspa@juntadeandalucia.es; 4 Instituto de Investigación Biosanitaria (ibs.GRANADA), 18012 Granada, Spain; jlrbejar@ugr.es; 5 Ceuta Faculty of Health Sciences, Campus Universitario de Ceuta, University of Granada, 51001 Ceuta, Spain; jlgurquiza@ugr.es; 6 Department of Statistics and Operations Research, University of Granada, Av. de Fuente Nueva, s/n, 18071 Granada, Spain; 7 Institute of Mathematics, University of Granada (IMAG), 18016 Granada, Spain; 8 Brain, Mind and Behaviour Research Center (CIMCYC), University of Granada, 18071 Granada, Spain; 9 Department of Didactic of Mathematics, Faculty of Education, University of Granada, Campus Universitario de la Cartuja, 18071 Granada, Spain; grcanadas@ugr.es

**Keywords:** burnout, latent profile analysis, nursing, psychology, students

## Abstract

There is abundant literature suggesting that university students in helping professions experience high levels of stress, leading to an increased risk of developing burnout. The objective of this study was to identify burnout profiles in a sample of 1162 Spanish nursing and psychology undergraduates using latent profile analysis, a person-oriented statistical method that can identify hidden homogenous subgroups within a heterogeneous population. We expected to replicate in university students the five-profile structure (burnout, overextended, disengaged, ineffective, and engagement) proposed by Leiter and Maslach using the burnout dimensions (emotional exhaustion, cynicism, and inefficacy) as indicators. The results showed that burnout, overextended, and engagement profiles were adequately replicated. Given that levels of inefficacy and cynicism were medium to low, the ineffective and disengaged profiles somewhat deviated from those identified by Leiter and Maslach. We found differences between the five latent profiles in several psychological variables, such as depression and anxiety. These results suggest that psychosocial factors (e.g., workload) are significant among students and may adversely impact their health, leading to psychosomatic and emotional disorders. Hence, designing effective interventions to prevent health problems associated with burnout seems advisable, considering the specific burnout profile that a student exhibits.

## 1. Introduction

There is abundant literature suggesting that college students experience high levels of stress [1,2,3,4,5,6,7,8,9], with some evidence of increases following the events of the COVID-19 pandemic [10]. According to the traditional model proposed by Maslach and colleagues [11,12,13], burnout is a psychological syndrome emerging as a prolonged response to chronic interpersonal stressors on the job, classically described on three interrelated dimensions: emotional exhaustion, depersonalization (or cynicism), and low personal accomplishment (or inefficacy). Since 2019, burnout has been included in the WHO International Classification of Diseases, impacting both psychological and physical well-being [14,15]. Its effects on students are varied [2], encompassing psychosomatic issues (such as gastrointestinal problems, sleep disturbances, and fatigue), emotional challenges (including depression and demotivation), and behavioral issues (such as declining academic performance, absenteeism, and dropout rates).

Based on Maslach’s theoretical framework, Schaufeli et al. [8] proposed that, among students, burnout refers to feeling exhausted because of study demands, having a cynical and detached attitude toward study, and feeling inefficacy as a student. In this context, emotional exhaustion denotes the sense of stress encountered in the academic setting, specifically addressing the persistent fatigue that can arise due to an overwhelming academic workload. Cynicism manifests as a lack of concern or detachment from school-related tasks, a waning enthusiasm for one’s own academic endeavors, and a perception of studying as devoid of purpose. Inefficacy pertains to the perception of diminished effectiveness in both studying and academic accomplishments, as well as a sense of devaluation in the tasks undertaken and in the overall school experience. A combination of high levels of emotional exhaustion, cynicism, and inefficacy is considered to be indicative of burnout [12,13].

Traditional research on burnout has shown several associations between the three dimensions, for instance, moderate positive correlations between emotional exhaustion and cynicism, and weak to moderate negative correlations between inefficacy and the other two dimensions [3]. The distinct associations between the three dimensions of burnout may reflect their different developmental order [16]. In this vein, several models of the onset of burnout have been proposed, such as the one by Maslach and colleagues [17], that suggests exhaustion appears in the initial stage, followed by cynicism, with inefficacy occurring later. In contrast, Golembiewski’s model [18] proposed that emotional exhaustion arises in the final stage, starting with cynicism symptoms. While certain research efforts have explored the causal connections between the three dimensions of burnout, the findings have not consistently aligned with the original model’s propositions [19]. 

Nevertheless, research has shown that the experience of burnout may differ from one individual to another with respect to the different dimensions of burnout [20,21]. In this sense, Leiter and Maslach [22] proposed a shift towards a person-centered approach rather than a variable-centered one to characterize the internal organization of burnout symptoms more effectively within individuals.

### 1.1. Person-Centered Approach and Latent Profile Analysis

In contrast to variable-centered approaches, such as structural equation modeling, traditional burnout classifications, which examine relationships between variables, and person-centered methods, like latent profile analyses, seek to uncover cohesive subgroups within a diverse population [23,24]. In contrast to conventional clustering methods, such as k-means clustering and hierarchical clustering, latent profile analysis (LPA) considers profile membership as an unobservable categorical variable; that is, it is a type of latent variable model that can be used to identify latent classes in a dataset, based on a set of continuous input variables [23,25,26]. 

LPA proves to be valuable when there is significant diversity among individuals in their scores across multiple variables, and when this variability cannot be accounted for by observable factors [27]. In essence, LPA operates on the assumption that by introducing a categorical latent variable, it can effectively reduce the residual variance within the sample, thus dividing the sample into two or more subgroups that exhibit greater homogeneity in terms of their patterns of variable means and variances/covariances.

The identified classes can exhibit different patterns of means that are specific to each class, as well as variances that are either class-specific or vary among classes, depending on the model specification. The models can be specified in terms of central tendency and variability of the indicators that form the classes, as well as in regard to the relationships among them.

Firstly, it is common to specify that the means vary freely within each class and between different classes, although it is also possible to restrict the model by specifying that they are equal. Secondly, it can be specified that the variances of each indicator can vary within each class and between classes, only within each class, or only between classes. Finally, model specifications include, on the one hand, that the covariances between the indicators within classes are set to zero (indicating no relationship between observed variables) or are estimated freely, and, on the other hand, that the covariances between the indicators can be different (or equal) between classes. Finally, model specifications include, on the one hand, the possibility to set the covariances between indicators within classes to zero, indicating no relationship between observed variables, or to estimate them freely. On the other hand, it also can be considered whether the covariances between indicators may vary or remain equal across classes. For a more detailed description of the different parameterizations in LPA, Scrucca et al. [28] can be consulted.

### 1.2. Research on Burnout Profiles

Employing LPA on two samples of health care workers, Leiter and Maslach [22] identified five burnout profiles: burnout (high scores on all three dimensions), engagement (low on all three), overextended (high on exhaustion only), ineffective (high on inefficacy only), and disengaged (high cynicism only). The prevalent profile observed was engagement, followed by ineffective individuals.

Based on these findings, other researchers have begun to study these latent profiles in different occupational groups, such as teachers, nurses, police officers, veterinarians, and researchers, using the Maslach Burnout Inventory (MBI) [11] as a measuring instrument [21,29,30,31,32,33,34,35]. The emerging research in working populations has, in most cases, failed to replicate the original five-profile structure. The most frequently observed solution has been that of four profiles [33,35], with one study on young researchers [29] successfully replicating the solution proposed by Leiter and Maslach [22]. However, the results of this study also demonstrated a good fit for a four-profile structure.

In the case of university students, we have only come across three studies that utilize the MBI to investigate latent profiles, and none of them has succeeded in replicating the five-profile structure originally identified by Leiter and Maslach [22]. Specifically, while two studies identified a three-profile solution [5]. In the first two studies, the profiles were similar, with two opposing profiles (burnout and engagement) and an intermediate one showing moderate levels of emotional exhaustion. In the study with the four-profile solution, similar profiles of burnout and engagement were identified, along with two profiles closely resembling each other (denominated as “moderate below-average burnout” and “moderate above-average burnout”).

Given the scarcity of research on this topic, the main objective of our study was to identify burnout latent profiles in a sample of Spanish nursing and psychology undergraduates using LPA. Drawing from the research conducted by Leiter and Maslach in [22] on employee burnout profiles, we anticipated the identification of five distinct profiles. Two profiles encompassed the extremes of the burnout spectrum, one characterized by high scores on all three MBI scales, signifying high burnout, and the other marked by low scores across all scales, indicating low burnout. The other three are mixed profiles with high scores on only one MBI scale: overextended, i.e., featured high emotional exhaustion scores along with medium scores on the other two scales; disengaged, i.e., displayed high cynicism scores exclusively; and ineffective, with high inefficacy scores as its distinctive characteristic.

The second objective was to investigate differences between the identified latent profiles on some psychological and sociodemographic variables that the research literature in the field has found to be associated with burnout syndrome. On the one hand, we selected personality traits that may act as risk factors, such as neuroticism, extraversion, conscientiousness, and agreeableness [1,36,37,38]. On the other hand, depression and anxiety were chosen as health outcomes resulting from experiencing burnout [39,40], which take a place in the job demands-resources model [41,42,43].

## 2. Materials and Methods

### 2.1. Participants

The sample consisted of 1162 nursing and psychology students from all academic years (from first to fourth) selected from different Spanish universities (Granada, Melilla, Ceuta, and Málaga) through non-probabilistic sampling. Among these participants, 64.7% were female, and the average age was 20.9 years (SD = 1.92).

### 2.2. Procedure

The data for the study were gathered in the initial quarter of 2019, conducted within the classroom setting and during official academic hours, with the consent and approval of the university staff. All participating students provided informed consent in advance, with assurances of confidentiality and anonymity.

### 2.3. Instruments

Every participant filled out a customized sociodemographic questionnaire, providing information on their age, gender, academic year, and grade. The subsequent measuring instruments were administered in their Spanish versions.

The Maslach Burnout Inventory-Student Survey (MBI-SS) [8] was used to measure burnout syndrome. This questionnaire contains 15 items scored on a seven-point response scale, to measure the following three dimensions of the syndrome stipulated in the original proposal by Maslach and Jackson [11]: emotional exhaustion, depersonalization (or cynicism), and low personal accomplishment (or inefficacy). The omega reliability values in the current sample were 0.78 for emotional exhaustion and 0.79 for cynicism as well as for inefficacy.

Four subscales of the NEO Five Factor Inventory (NEO-FFI) [44] were used to measure the traits neuroticism, extraversion, conscientiousness, and agreeableness. Each subscale consists of 12 items, scored on a five-point Likert response format. The omega reliability values in the present sample were 0.81 for neuroticism, 0.69 for agreeableness, 0.80 for conscientiousness, and 0.83 for extraversion.

The depression and anxiety dimensions of the Educational-Clinical Questionnaire: Anxiety and Depression (CECAD) [45] was employed to assess depression and anxiety. This questionnaire consists of 50 items with a five-point Likert-type response format. It produces a global evaluation of emotional disorders, based on the scores obtained for six dimensions. We used depression subscale (which also comprises uselessness, irritability, and problematic thoughts) and anxiety subscale (which is also composed with psychophysiological symptoms). The omega reliability values in the current sample were 0.86 for depression and 0.90 for anxiety.

### 2.4. Statistical Analysis

All statistical analyses were carried out in R 4.2.2 [46] using the mclust 6.0.0 [28] and tidyLPA 1.1.0 [47] packages for the latent profile analysis.

Missing values were handled using the listwise deletion method, as the percentage of missing data was less than 3%. The specification of the statistical model and the number of profiles was decided based on both statistical and substantive criteria. From a theoretical standpoint, one can expect the means of the indicators to vary across profiles, as well as the variances and covariances. However, if this is not the case in the sample, opting for a more parsimonious model will likely achieve a better fit to the data. For this reason, we compared different statistical specifications using the Bayesian information criteria (BIC) [48]. Estimation of the model parameters was undertaken using maximum likelihood (ML) estimation via the EM algorithm [49]. Several model specifications were tested; for example, the one with variable volume, shape, and orientation (VVV) and the one with variable volume and shape and coordinate axis orientation (VVI).

We assessed the models with the log-likelihood (LL), four global fit indices, two likelihood ratio tests, and two classification accuracy indices. Following recommendations in the field [23,50,51,52,53], the employed global fit indices were BIC, sample size-adjusted Bayesian information criterion (SABIC) [54], Akaike’s information criterion (AIC) [55], and the integrated completed likelihood (ICL). For the LL and all the global fit indices, values closer to zero reflect a better fitting of the model.

The following two likelihood ratio tests were used: Lo–Mendell–Rubin’s likelihood ratio test (Lo-LRT) [56] and the bootstrap likelihood ratio test (BLRT) [49], in which the model with G profiles is compared with the one with G-1, and a statistically significant result indicates that the extra profile is required.

The classification accuracy of each model was examined using the entropy statistic [57] and the average posterior probabilities. Both indices range from zero to one, with values closer to one being better. Entropy values greater than 0.8 and average posterior probabilities greater than 0.70 indicate a good classification accuracy of the model [58,59,60].

We additionally considered the interpretability, distinguishability, and sample size of the identified latent profiles. For example, we rejected models that contained small profiles (e.g., less than 1% or *n* = 25), as these profiles are typically spurious [61].

Finally, an analysis of variance was conducted with profiles as independent variables and psychological outcomes as dependent variables. Post hoc comparisons were performed using Tukey’s Honestly Significant Difference correction. Assumptions of normality, independence of observations, and homoscedasticity were checked. Since the assumption of homoscedasticity was not met, robust standard errors were used to account for the violation of this assumption. Partial eta squared was used as the effect size for the main effects. Likewise, chi-squared tests were used to check for differences in sociodemographic variables (age, gender, academic year, and grade) between profiles. Adjusted standardized residuals were analyzed using the Bonferroni correction for pairwise comparisons and Cramer´s V statistic was employed as the effect size.

## 3. Results

In the first place, descriptive variable-oriented statistics were calculated. The mean MBI dimension scores were 2.74 (SD = 1.24) for emotional exhaustion (EE), 1.71 (SD = 1.35) for cynicism (C), and 1.50 (SD = 0.94) for inefficacy (IN). Pearson correlation coefficients between dimensions were moderate to high, concretely, with 0.50 for EE and C, 0.20 for EE and IN, and 0.40 for C and IN.

**Figure 1:**
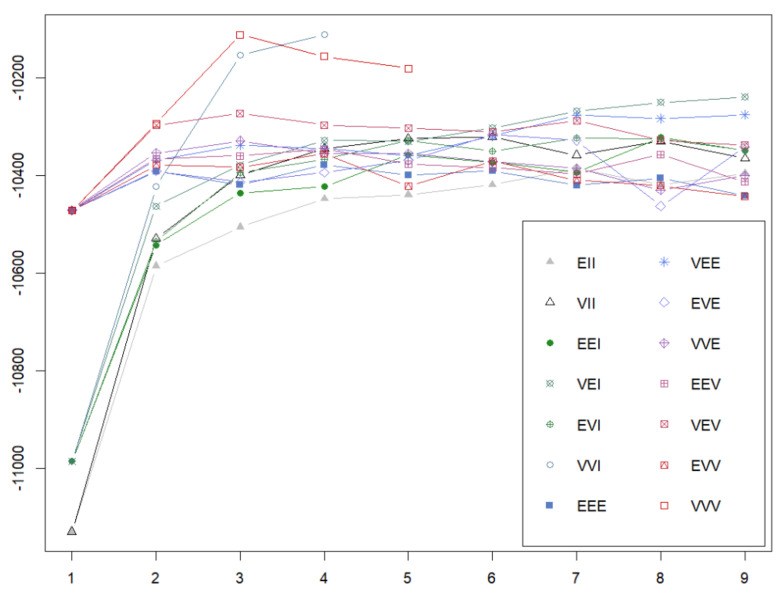
BIC results for the statistical models. Note: number of components (x axis) versus Bayesian information criteria (BIC) (y axis). Statistical models are represented by the following identifiers: VEI, EVI, VVI, EEE, VEE, EVE, VVE, EEV, VEV, EVV, and VVV, where the first, second, and third identifier refers to volume, shape, and orientation, respectively; E = equal, V = variable; I = coordinate axes.

In the second place, person-oriented approach analyses were performed. In this regard, while our initial expectation was to replicate the five-profile model identified by Leiter and Maslach [22], we conducted successive exploratory models, ranging from one to six components, incorporating various statistical specifications (e.g., VVI, VVV), in order to check which model best fit the data. Figure 1 illustrates that the data favored (the closer the BIC values are to zero, the better) a VVI specification with four profiles (BIC = 10,110.50), closely followed by a VVV specification with three profiles (BIC = 10,110.69).

In agreement with these results, we decided to use the VVI and VVV statistical models to determine the number of latent profiles. As depicted in Table 1, global fit indices showed inconsistent findings: although BIC indicated that the VVI four-profile solution was the best model, AIC, ICL, and SABIC demonstrated a better fit for the VVV five-profile model. The solutions for the VVI five-profile and VVV six-profile models did not converge, indicating that these models and models with more profiles should not be retained. The findings from both the bootstrapped likelihood ratio test and the Lo–Mendell–Rubin’s adjusted likelihood ratio test were consistent, suggesting the retention of the four-profile structure for the VVI model and the five-profile structure for the VVV model. Entropy values, which indicate the overall competence of a model to return well-separated profiles, were satisfactory for all the models, ranging from 0.71 to 0.92.

Table 2 and Table 3 show the profile average posterior probabilities and the profile size for all the models. The average posterior probabilities express the likelihood that cases will be accurately categorized into the appropriate profile rather than an incorrect one. Probabilities for all the profiles in all models were above the recommended cut-off value (0.70). Regarding profile size, all the profiles accumulated more than 3% of the sample, indicating substantive profiles.

**Figure 2:**
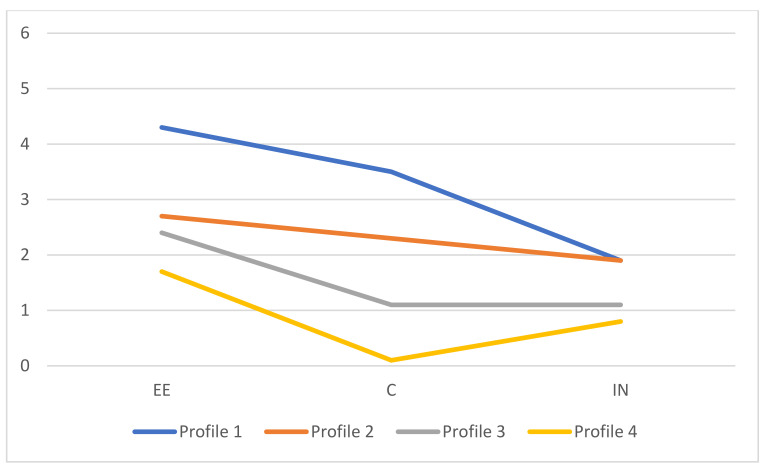
Profiles means for model VVI with four components. Note: profile 1 = “exhausted and disengaged”; profile 2 = “at risk of burnout”; profile 3 = “at risk of overextended”; profile 4 = “engagement”.

**Figure 3:**
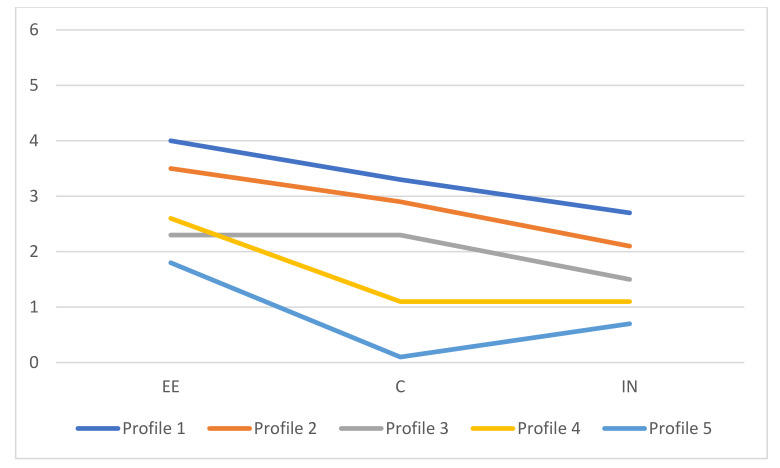
Profiles means for model VVV with five components. Note: profile 1 = “burnout”; profile 2 = “overextended”; profile 3 = “disengaged”; profile 4 = “at risk of overextended”; profile 5 = “engagement”.

Based on these statistical results, the best models to choose were the four-profile solution with VVI specification and the five-profile solution with VVV specification. Therefore, we proceeded to examine the interpretability of the profiles. Figure 2 and Figure 3 provide a graphical representation of the estimated means for both solutions. While doing so, we endeavored to adhere to the nomenclature employed by Leiter and Maslach [35].

In the solution with four profiles and the VVI specification, profile 1 was termed “exhausted and disengaged” as the means were high for EE and C, and low for IN. Profile 2 was labelled “at risk of burnout”, given that the means were moderated in all dimensions. Profile 3 was named “at risk of overextended”, as the mean for EE was moderate and the means for C and IN were low. Finally, profile 4 was termed “engagement”, given that the means were low in all dimensions.

In the solution with five profiles and the VVV specification, profile 1 was named “burnout”, as the means in all dimensions were moderate to high. Profile 2 was labelled “overextended”, given that the mean for EE was high, and the means for C and IN were from low to moderate. Profile 3 was termed “disengaged”, as the means for EE and C were moderate and low for IN. Profile 4 was named “at risk of overextended”, as the mean for EE was moderate and the means in the other two dimensions were low. Finally, profile 5 was termed “engagement”, given that the means were low in all dimensions.

Considering the interpretability of the profiles alongside the statistical findings, we contend that the optimal solution is the model featuring five latent profiles with the VVV specification. Despite one profile representing only 4.6% of the sample, its inclusion is justified as it is neither redundant nor falls below the cut-off values of 1% and 3%, as suggested by Lubke and Neale [61] respectively. Furthermore, the profiles in the VVV solution closely align with the five profiles previously identified by Leiter and Maslach [22].

Using this model, an analysis of variance (ANOVA) was conducted, with the profile as the independent variable and various psychological variables (depression, anxiety, neuroticism, extraversion, conscientiousness, and agreeableness) as dependent variables. All main effects were statistically significant with high effect sizes in most situations, indicating that there were relevant differences between the profiles in these variables. Concretely, ANOVA results were F(4, 1046) = 42.06, *p* < 0.05, η^2^ = 0.164 for depression, F(4, 1046) = 47.39, *p* < 0.05, η^2^ = 0.191 for anxiety, F(4, 1046) = 34.05, *p* < 0.05, η^2^ = 0.114 for neuroticism, F(4, 1046) = 34.31, *p* < 0.05, η^2^ = 0.127 for extraversion, F(4, 1046) = 32.82, *p* < 0.05, η^2^ = 0.109 for conscientiousness, and F(4, 1046) = 31.20, *p* < 0.05, η^2^ = 0.077 for agreeableness.

Post hoc pairwise comparisons are shown in Table 4. Statistically significant differences were observed between every pair of profiles in at least one dependent variable, with extraversion exhibiting the highest number of differences and agreeability showing the fewest. Students in the “burnout” profile exhibited higher levels of depression, anxiety, neuroticism, and introversion compared to students in the other profiles. Additionally, they demonstrated lower levels of agreeableness than students in the “disengaged” profile and lower conscientiousness than students in both the “at risk of overextended” and “engagement” profiles. Students in the “disengaged” profile displayed lower levels of extraversion, conscientiousness, and agreeableness than students in the “at risk of overextended” and “engagement” profiles. Finally, the only statistically significant difference identified between the “at risk of overextended” and “engagement” profiles pertained to conscientiousness, with students at risk of overextension exhibiting lower levels of this variable than engaged students.

Regarding sociodemographic variables, while no statistically significant differences were found between nurses and psychologists (χ^2^ = 8.45, *p* = 0.076, V = 0.06) neither in age (F(4, 1046) = 0.89, *p* = 0.569, η^2^ = 0.003), we found them in gender (χ^2^ = 54.77, *p* < 0.05, V = 0.23) and in academic year (χ^2^ = 32.03, *p* < 0.05, V = 0.10). On the one hand, there were more male students pertaining to “overextended” and “disengaged” profiles than female students. Additionally, there were more female students in “at risk of overextended” and “engagement” profiles than male students. On the other hand, there were more second-year students in the profile “engagement” than students in other academic years.

## 4. Discussion

The goal of this study was to examine latent burnout profiles in a sample of nursing and psychology students, based on the framework proposed by Leiter and Maslach [22]. This framework identifies five profiles within the burnout–engagement continuum: burnout, overextended, disengaged, inefficacy, and engagement. To achieve this, latent profile analysis [23,24,26,62] was employed, a novel statistical technique with notable advantages. Firstly, it focuses on individuals’ behavioral tendencies rather than mere variable associations. Secondly, it works with latent variables, meaning that the resulting groups refer to profiles that are not directly observable.

We identified a structure with five latent profiles, of which four were consistent with the proposal by Leiter and Maslach [22]. The clearest similarities were found in the two extreme profiles: burnout and engagement. Concerning the former, a profile was identified with high levels of emotional exhaustion and cynicism and intermediate levels of inefficacy. Regarding the latter, the levels of all three dimensions were low in this profile. One, or both, of these profiles have been replicated in the majority of studies using the MBI as an instrument to assess burnout, both in students [5,63,64] and in workers [29,30,31,32,34,35,65].

The profile referred here to as “overextended” aligned more closely with the findings of the second study than the first by Leiter and Maslach [22]. In the current study, it exhibited high levels of emotional exhaustion, intermediate levels of cynicism, and low levels of inefficacy. This profile, where levels of emotional exhaustion and cynicism are elevated or moderate, has been identified in other studies involving workers [31], and it may suggest a profile not originally hypothesized in the Leiter and Maslach [22] model. However, in a study by Portoghese et al. [5,63,64], the overextended profile was replicated appropriately, as cynicism levels were low. The profiles that showed the most discrepancies were “disengaged”, with moderate levels of cynicism and emotional exhaustion and low levels of inefficacy, and the profile “at risk of overextended”, which exhibited moderate levels of emotional exhaustion and low levels in the other two dimensions. The profile that was not replicated in this study was the one referred to as ineffective, given that students in the sample generally had low levels of inefficacy. The results of other studies with students have also failed to replicate these intermediate profiles characterized by high levels in a single dimension [63]. Similarly, in the case of workers, one or several of these profiles have not been frequently [26,29,30,31].

Regarding the prevalence of the profiles, the most common was overextended (35.9%), followed by disengaged (26.2%), at risk of overextended (18%), engagement (15.4%), and finally, burnout (4.6%). These findings contrast with those of Leiter and Maslach [22], where the most prevalent profiles were ineffective and engagement. In other studies conducted with students, consistency has not been observed either. For instance, the engagement profile is around 15–18% of the sample, and the burnout profile ranges from 11% to 34% [5,63,64]. It is important to note that, in these studies, the structure of five profiles was not replicated, and therefore, discrepancies in percentages may be attributed to the smaller number of profiles. Additionally, in a study that successfully replicated the structure of five profiles, discrepancies in profile prevalence were observed [29].

The fact that differences were detected among the five latent profiles in psychological variables provides evidence of criterion validity for the obtained structure [23,53]. As expected, students in the burnout profile exhibited higher levels of depression, anxiety, neuroticism, and introversion than those in the other profiles. Specifically, they showed lower levels of agreeableness and conscientiousness than students in the engagement and at risk of burnout profiles. In contrast, students in the engagement profile displayed the opposite pattern, with lower levels of depression, anxiety, neuroticism, extraversion, conscientiousness, and agreeableness than students in the burnout, overextended, and disengaged profiles. Regarding the at risk of burnout and engagement profiles, differences were only observed in agreeableness and conscientiousness.

These differences in the latent profiles have implications for both psychological theory and clinical practice. In terms of psychological theory, these results align with the job demands-resources theory, which hypothesizes that individuals with burnout will have higher levels of depression and anxiety than engaged ones [41]. As Leiter and Maslach [22] point out, these results suggest that burnout is a phenomenon that is not strictly analogous to emotional exhaustion or the combination of emotional exhaustion with cynicism [66,67]. Furthermore, these findings suggest that the cynicism dimension plays a relevant role independently of the other dimensions, as disengaged was the second most prevalent profile and showed differences in psychological variables compared to other profiles such as burnout and overextended.

Regarding practical implications, intermediate profiles with only one high dimension can serve as indicators that an individual is at risk of developing burnout, prompting the implementation of preventive strategies. In this regard, individuals in the overextended profile would benefit from interventions focused on improving depressive and anxiety symptoms to a greater extent than students in the disengaged profile. Additionally, according to our results, early intervention programs should target neuroticism, extraversion, and agreeableness, as no differences in conscientiousness were found between these two profiles. Additionally, we found that male students tend to be in risky burnout profiles, such as overextended and disengaged. Hence, it seems reasonable that initial assessment of burnout symptoms does not neglect these students, who typically constitute a low percentage of the global student population in these academic grades.

### Limitations and Future Research

A limitation of the current study is the use of a cross-sectional design. It would be highly interesting for future research to conduct a longitudinal study that analyses the development of latent burnout profiles through repeated measures latent class analysis [23].

Another limitation is that this study only utilized predictor variables of a psychological nature. These predictor variables are crucial for verifying whether latent profiles are associated with different risk factors or health outcomes. Future studies should expand on the work conducted here by investigating work-related and academic variables using a substantive psychological framework such as the job demands-resources theory [41,42,43].

Finally, in this work, engagement, considered as the opposite pole of burnout, has not been measured. It would be interesting for future studies to include this variable in a latent profile analysis to observe the types of profiles that emerge.

## 5. Conclusions

Latent profile analysis is a person-oriented statistical method that can identify hidden homogenous subgroups within a heterogeneous population. It is a sophisticated method that is starting to receive attention in applied psychology to complement traditional variable-oriented methods. Using nursing and psychology undergraduates as participants, our aim was to reproduce the five-profile structure (burnout, overextended, disengaged, ineffective, and engagement) outlined by Leiter and Maslach [22], employing the burnout dimensions (emotional exhaustion, cynicism, and inefficacy) as observable indicators.

We successfully reproduced a five-profile structure, with four profiles aligning closely with the framework proposed by Leiter and Maslach [22]. The most consistently observed profiles were burnout and engagement, characterized by high and low levels, respectively, across all three dimensions. The overextended and disengaged profiles also demonstrated consistent patterns, with the former exhibiting elevated exhaustion and the latter displaying moderate levels of cynicism and exhaustion. Notably, the ineffective profile outlined by Leiter and Maslach [22] did not emerge in our study, as students in our sample generally exhibited low levels of inefficacy.

We observed significant differences in psychological variables among the five latent profiles. Concretely, students in the burnout profile demonstrated elevated levels of depression, anxiety, neuroticism, and introversion compared to their counterparts in the other profiles. Conversely, students in the engagement profile exhibited the opposite trend. Also, male students showed higher levels of exhaustion and cynicism than female students. These findings hold practical implications for clinicians and stakeholders, given that it seems advisable to design interventions in accordance with the specific burnout profile that a student exhibits. For instance, male students, who tend to be in the overextended profile, may benefit from interventions aimed at ameliorating depressive and anxiety symptoms. Furthermore, early intervention programs should strategically address factors such as neuroticism, extraversion, and agreeableness.

## Figures and Tables

**Table 1:** Model fit results from latent profile analysis.

Model	Components	Log-Likelihood (df)	ICL	AIC	BIC	SABIC	Entropy	BLRT ^a^	Lo-LRT ^a^
VVI	3	−5005.708 (20)	−10,811	10,364.60	10,152.57	10,391	0.71	319.024 *	-
VVI	4	−4959.969 (27)	−10,961	10,331.29	10,110.50	10,365	0.68	91.478 *	87.35 *
VVI	5	-	-	-	-	-	-	-	-
VVV	3	−4953.007 (29)	−10,811	10,364.60	10,110.69	10,391	0.72	252.649 *	-
VVV	4	−4940.123 (39)	−10,961	10,331.29	10,155.50	10,365	0.68	25.768 *	24.61 *
VVV	5	−4917.035 (49)	−10,749	10,245.66	10,179.91	10,287	0.79	46.175 *	44.09 *
VVV	6	-	-	-	-	-	-	-	-

Note: VVI = diagonal, varying volume and shape; VVV = ellipsoidal, varying volume, shape, and orientation; ICL = integrated completed likelihood; AIC = Akaike’s information criteria; BIC = Bayesian information criteria; SABIC = sample size-adjusted BIC; BLRT = bootstrapped likelihood ratio test; Lo-LRT = Lo–Mendell–Rubin’s adjusted likelihood ratio test. ^a^ BLRT and Lo-LRT compare the indicated model (G components) to a model with G-1 components. Statistical significance indicates that the indicated model (G components) yields a better fit than the model with G-1 components. * = p < 0.05.

**Table 2:** Average profile posterior probabilities according to the statistical model.

Model	Components	Profile 1	Profile 2	Profile 3	Profile 4	Profile 5
VVI	3	0.84	0.86	0.91	-	-
VVI	4	0.79	0.86	0.79	0.82	-
VVI	5	-	-	-	-	-
VVV	3	0.84	0.80	0.92	-	-
VVV	4	0.84	0.81	0.71	0.75	-
VVV	5	0.84	0.74	0.78	0.83	0.72
VVV	6	-	-	-	-	-

Note: VVI = diagonal, varying volume and shape; VVV = ellipsoidal, varying volume, shape, and orientation.

**Table 3:** Profile size according to the statistical model.

Model	Components	Profile 1		Profile 2		Profile 3		Profile 4		Profile 5	
		%	N	%	N	%	N	%	N	%	N
VVI	3	41.8	486	16.4	190	41.8	486	-	-	-	-
VVI	4	34.3	398	16.2	189	33.3	387	16.2	188	-	-
VVI	5	-	-	-	-	-	-	-	-	-	-
VVV	3	18.1	210	34.9	406	46.9	546	-	-	-	-
VVV	4	18.3	213	36.0	418	27.1	315	18.6	216	-	-
VVV	5	18.0	208	15.4	179	35.9	417	4.6	54	26.2	304
VVV	6	-	-	-	-	-	-	-	-	-	-

Note: VVI = diagonal, varying volume and shape; VVV = ellipsoidal, varying volume, shape, and orientation.

**Table 4:** Pairwise comparisons between profiles on psychological variables.

Profiles Comparison	Depression		Anxiety		Neuroticism		Extraversion		Conscientiousness		Agreeability	
	MD	*t* ^a^	MD	*t*	MD	*t*	MD	*t*	MD	*t*	MD	*t*
1–2	11.5	5.05 *	8.6	6.09 *	4.9	4.88 *	−2.6	2.83 *	0.9	1.17	−0.2	−0.24
1–3	22.9	9.87 *	15.9	11.07 *	8.7	8.40 *	−5.1	5.42 *	0.1	0.13	−1.4	−1.72
1–4	22.9	10.15 *	15.7	11.25 *	8.5	8.47 *	−7.5	8.19 *	−2.5	−3.42 *	−3.3	−4.20 *
1–5	25.8	10.91 *	18.1	12.37 *	10.0	9.51 *	−3.0	4.63 *	−3.9	−5.10 *	−3.9	−4.70 *
2–3	11.4	7.73 *	7.3	8.25 *	3.7	5.77 *	−2.5	4.21 *	−0.7	−1.61	−1.6	−3.12 *
2–4	11.4	8.30 *	7.1	8.68 *	3.5	5.90 *	−4.9	8.87 *	−3.3	−7.69 *	−3.5	−7.40 *
2–5	14.3	9.26 *	9.5	10.23 *	5.1	7.44 *	−5.5	8.78 *	−4.7	−9.64 *	−4.1	−7.70 *
3–4	0.1	0.01	0.02	0.18	0.2	0.31	−2.4	4.17 *	−2.6	−5.67 *	−1.9	−3.89 *
3–5	2.9	1.84	2.2	2.32	1.3	1.85	−3.0	4.63 *	−4.0	−7.80 *	−2.5	−4.58 *
4–5	2.3	1.96	2.4	2.64	1.5	2.26	−0.6	0.97	−1.4	−2.92 *	−0.6	−1.18

Note. 1 = “burnout” profile; 2 = “overextended” profile; 3 = “disengaged” profile; 4 = “at risk of overextended” profile; 5 = “engagement” profile. MD = mean difference; *t* = *t*-value; ^a^ = degrees of freedom for each test are 1046. * = p < 0.05.

## Data Availability

The datasets generated and/or analyzed during the current study are not publicly available due to respondents’ confidentiality but are available from the corresponding author on reasonable request.
